# Photopolymerized keratin-PGLa hydrogels for antibiotic resistance reversal and enhancement of infectious wound healing

**DOI:** 10.1016/j.mtbio.2023.100807

**Published:** 2023-09-23

**Authors:** Changfa Sun, Wenjie Liu, Lili Wang, Run Meng, Jia Deng, Rui Qing, Bochu Wang, Shilei Hao

**Affiliations:** a Key Laboratory of Biorheological Science and Technology, Ministry of Education, College of Bioengineering, Chongqing University, Chongqing, 400030, China; b College of Environment and Resources, Chongqing Technology and Business University, Chongqing, 400067, China; c State Key Laboratory of Microbial Metabolism, School of Life Sciences and Biotechnology, Shanghai Jiao Tong University, Shanghai, 200240, China

**Keywords:** Keratin, Photopolymerizable hydrogels, Infectious wound healing, Antibiotic resistance reversal

## Abstract

Infectious wounds have become serious challenges for both treatment and management in clinical practice, so development of new antibiotics has been considered an increasingly difficult task. Here, we report the design and synthesis of keratin 31 (K31)-peptide glycine-leucine-amide (PGLa) photopolymerized hydrogels to rescue the antibiotic activity of antibiotics for infectious wound healing promotion. K31-PGLa displayed an outstanding synergistic effect with commercial antibiotics against drug-resistant bacteria by down-regulating the synthesis genes of efflux pump. Furthermore, the photopolymerized K31-PGLa/PEGDA hydrogels effectively suppressed drug-resistant bacteria growth and enhanced skin wound closure in murine. This study provided a promising alternative strategy for infectious wound treatment.

## Graphical abstract

**Figure:**
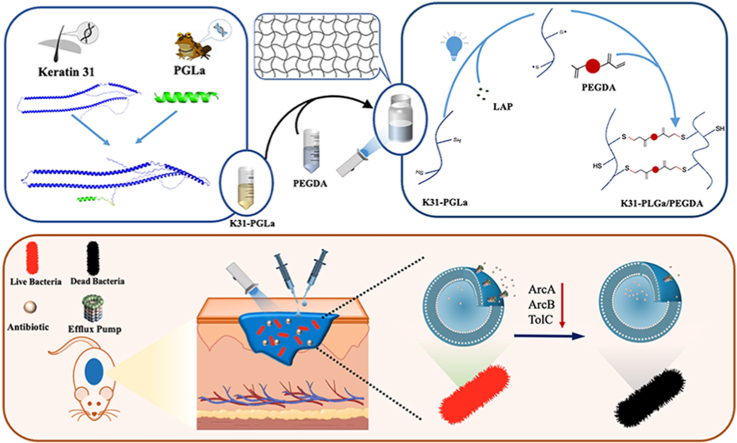

## 1 Introduction

Wound healing has always been one of the most serious issues faced by the medical community because the skin is easily damaged by diseases, fire, and mechanical accidents. Besides, rapid and effective wound healing is crucial for reducing significant morbidity and mortality rates, as well as alleviating financial burdens [[1], [2], [3], [4]]. Undoubtedly, wounds are easily suffered from bacterial infection, which has become serious challenges in the clinical practice, thereby increasing the risk of morbidity and mortality [5,6]. Moreover, drug-resistant bacteria cause more difficulty in infection treatment largely due to antibiotic abuse [7]. Although various therapeutic strategies, including the usage of novel antibacterial substances [[8], [9], [10]] and combined treatments [11,12], have been developed in the past decades, the secondary failure of antidiabetic therapies resulted in the limited outcomes of infectious wounds treatments [13]. Therefore, development of more efficient therapies for infectious wound in clinical practice is desperately needed.

The development of new antibiotics is considered to be an important way to overcome antibiotic resistance [[14], [15], [16]]. Only about 30–40 new antibacterial candidates are currently being tested in the clinical trial phases [17], but many pharmaceutical researches have failed to meet the clinical need for new antibiotics [18]. More seriously, only a tiny fraction of new antibiotics candidates was approved over the past 40 years, and the recent class of antibiotics was discovered during the 1980s [17,19]. Thus, it is essential to develop alternative therapeutic strategies for solving the difficulty [20,21]. Reversing antibiotic resistance would be a potential approach to address the antibiotic resistance threat. Multidrug evolutionary strategies have been suggested to reverse antibiotic resistance, since combining antibiotics is effective to inhibit specific resistance mechanisms due to the potential sensitivity of bacteria to another drug [22]. Conversely, enhancing sensitivity of antibiotic-resistant bacteria is an alternative strategy to reverse antibiotic resistance [23]. The polymer of pEt_20 was developed to reverse antibiotic resistance phenotype in MDR Gram-negative *A. baumannii*, and the polymer/antibiotic combination enabled the MIC to drop by 512-fold for both rifampicin and auranofin [24]. Besides, some Chinese herbal ingredients have been used as sensitizers to improve the sensitivity of antibiotic-resistant bacteria to medical antibiotics [25]. In addition, hydrogels with a 3D hydrophilic polymer structure are widely used in wound dressings because they provide protective barriers, simulate the primary extracellular matrix (ECM), and provide a humid environment [26]. Some functional molecules such as polymers, biological macro-molecules, and small chemical molecules can endow hydrogels with other special functions such as growth promotion, antibacterial, etc [27,28].

**Fig. 1:**
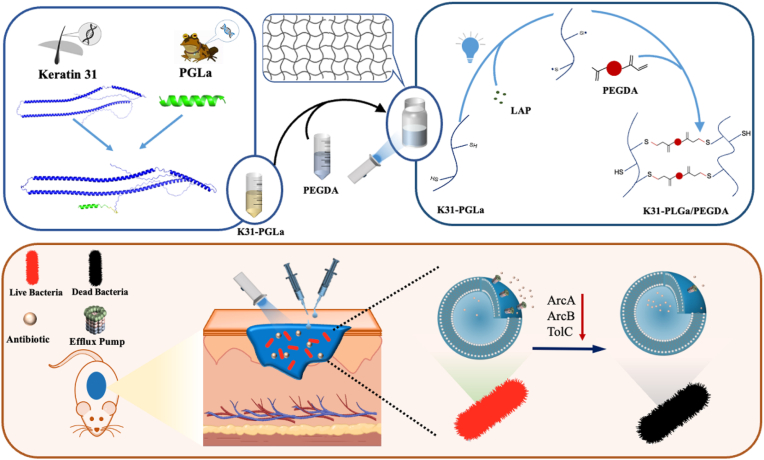
Schematic illustration of photopolymerized keratin-PGLa hydrogels preparation and reversal of resistant bacterial infection in skin wound.

Here, we report a kind of photopolymerized keratin-peptide glycine-leucine-amide (PGLa) hydrogels for antibiotic resistance reversal and enhancement of infectious wound healing. PGLa, a 21 amino acid residue peptide, is an antimicrobial peptide produced by the African clawed frog Xenopus laevis [29,30]. More importantly, antibiotic-PGLa pairs showed strong synergism in antibiotic resistance, which significantly slowed down the evolution of antibiotic resistance [31]. Furthermore, keratins have drawn increasing attention as promising candidates for wound healing, which is attributed to their excellent biocompatibility, broad sources, and highly biological actives for enhancing cell growth and migration [32,33]. Compared to the keratin extracts the superior wound repair efficiency of recombinant keratins was verified due to their higher purification in our previous study [34,35]. Keratin-based hydrogels showed great potential in the treatment of full-thickness skin wounds [[36], [37], [38]], diabetes skin wounds [39], and gastric ulcer wounds [40]. Therefore, the combination of keratin and PGLa would rescue the antibiotic activity of commonly used antibiotic drugs against resistant bacteria and enhance the infectious wound healing. To address this hypothesis, keratin 31 (K31), one of the most abundant keratins in human hair [41], was selected to fuse the PGLa for the infectious wound treatment combined with a common antibiotic drug. The photopolymerized keratin-PGLa/PEGDA composited hydrogels were synthesized for the in-situ wound therapy in this study, which would be rapidly gelled at desired locations for precise placement and effective filling of the injury site [42,43]. The physicochemical properties and infectious wound healing efficiency of developed keratin-PGLa/PEGDA hydrogels were assessed. Our results demonstrated that the photopolymerized keratin-PGLa hydrogels effectively suppressed drug-resistant bacteria growth and significantly enhanced skin wound closure in murine (Fig. 1).

## 2 Experimental

### 2.1 Protein expression and purification

The sequence information of keratin K31 (K31) was obtained from UniProt: https://www.uniprot.org/, and the sequence antimicrobial peptide PGLa was obtained from the previous report [29]. The fusion protein gene was amplified by whole gene synthesis PCR, connected to the pET-22b (+) vector (Fig. S1), and sequenced for verification. The recombinant protein was expressed in *E. coli* BL21 (DE3). Bacteria were cultured in an LB liquid medium containing 50 μg/mL ampicillin at 37 °C and 170 rpm. Isopropyl-***β***-D-thiogalactoside (IPTG) with a final concentration of 0.2 mM was added when OD_600_ reached 0.6–0.8. Bacteria were cultured at 16 °C and 160 rpm for 8 h, and then bacteria pellets were collected by centrifugation at 8000 rpm for 5 min at 4 °C. Bacteria were resuspended with 10 times the volume of buffer A (50 mM Tris-HCl, PH = 8; 150 mM NaCl; 1% Tween-20), and lysed by homogenizer (FB-110, Litu Machinery Equipment Engineering Co., Ltd. China). Inclusion bodies were collected by centrifugation (12000 rpm, 10 min, 4 °C). The inclusion bodies were washed with buffer B (50 mM Tris-HCl pH 8.0; 150 mM NaCl; 5 mM EDTA; 10 mM ***β***-mercaptoethanol; 1 mM urea; 0.5% TritonX-100) and buffer C (50 mM Tris-HCl pH 8.0; 2.5 M NaCl; 5 mM EDTA; 10 mM ***β***-mercaptoethanol). The washed inclusions were dissolved overnight with buffer D (50 mM Tris-HCl, pH 8.0, 150 mM NaCl, 8 M urea, 10 mM ***β***-mercaptoethanol). Insoluble precipitates were removed by centrifugation (12000 rpm, 20 min, 4 °C). The protein was purified by Ni-beads (Ni Smart Beads 6FF, Changzhou Tiandi Renhe Biotechnology Co., Ltd. China). The buffer D containing 0 mM, 10 mM and 20 mM imidazole were used successively to wash the Ni-beads to remove non-specific binding proteins. The K31-PGLa protein was then eluted twice with buffer D containing 250 mM imidazole. The purified protein was preserved after dialysis and freeze-drying.

### 2.2 Characterizations of K31-PGLa

Electrophoretic separations were performed on 12% w/v polyacrylamide separating gel using Bio-Rad Mini-Protean system to assess the molecular weight of proteins. The gel was placed in Coomassie Brilliant Blue for 30 min and then decolorization for 12 h, and the gel imager was used to observe and take pictures. The secondary structures of K31 and K31-PGLa were analyzed by a circular dichroism spectrophotometer (Chirascan, Applied Photophysics, UK). K31-PGLa and K31 were analyzed at 1 mg mL^−1^ with a scanning wavelength range of 190–280 nm at a rate of 100 nm min^−1^, spectral resolution of 1 nm, and test temperature of 25 °C. Fourier transform infrared spectrometer (Nicelet 6700, ThermoFisher, USA) was used to detect the characteristic absorption of chemical bonds. Freeze-dried protein samples were mixed with potassium bromide, ground into powder and pressed, and detected with FTIR spectroscopy (NicoletiS50, ThermoFisher, USA) in the wavelength range of 400–4000 cm^−1^.

### 2.3 Drug-resistant bacteria evolution

The method of laboratory evolution was used to obtain drug-resistant strains (*E. coli* and *S. aureus*), and the specific steps were carried out as follows [44]. Firstly, the strain was inoculated in antibiotic medium with decreasing concentration of 2 times. The previous concentration of the minimum inhibitory concentration (MIC) was selected as the initial concentration in the evolution process. Secondly, 2.5 mL of sterilized MHB liquid medium was added to the sterile 6-well plates, and 1% (25 μL) of bacteria with the initial concentration was subsequently added into the 6-well plates. Thirdly, the concentration of antibiotics was increased by 1.5 times every 2 days, and the passage was continued for 30 days. After 30 days of evolution, the MIC value was measured by Ultraviolet–visible spectrophotometer according to the OD_600_ value at each concentration, and the bacteriostasis rate was calculated according to the following formula (1). The logistic function was used to calculate the dose-effect relationship curve, proving the successful evolution of drug resistance.

(1) $$\text{Antibacterial}\;\text{rate}\left(\%\right)=\frac{A_{p}-A_{t}}{A_{p}-A_{n}}\times 100\%$$

*A*_*p*_ is the positive control (absorbance of media without antibiotics), *A*_*n*_ is the negative control (absorbance of media without antibiotics and strains), and *A*_*t*_ is the absorbance of the experimental group.

### 2.4 Antibacterial test

The antibacterial activities of K31, K31-PGLa, antibiotics, and the combination of protein and antibiotics on native *E. coli* and *S. aureus and* drug-resistant strains were assessed based on the MIC test. The optical density at 600 nm (OD_600_) of bacterial suspension treated with different proteins and antibiotics was detected, and the bacteriostasis rate was also calculated according to formula (1).

### 2.5 Quantitative real-time polymerase chain reaction (RT-PCR) analysis

Inoculate drug-resistant bacteria into two bottles of MHB liquid culture medium and culture in a shaker at 37 °C and 170 rpm until OD_600_ = 0.8. The first bottle only contains the antibiotic ampicillin, while the second bottle contains ampicillin and K31-PGLa. After 4 h of cultivation, collect bacterial precipitates by centrifugation. The total RNA of bacteria was extracted by the trizol method. cDNA was obtained by reverse transcription using the PrimeScript TM RT Reagen Kit. According to the TB Green Premix Ex *Taq*II (Tli RNaseH Plus) kit method, the CFX96 real-time PCR detection system was used for real-time fluorescence quantitative analysis. The primer sequences are listed in Table S1.

### 2.6 Cell counting kit-8 (CCK-8) assay

Hacat cells and L929 cells were used for protein cytotoxicity analysis. 5 × 10^4^ cells in each well were inserted into a 96-well plate and cultured for 24 h. Fusion protein solutions with different concentrations of 100, 200, 300, 400 and 500 μg/mL were configured according to the concentration gradient. 10 μL of fusion protein K31-PGLa was added to each well. After 24 h of incubation, 10 μL of CCK-8 solution was added. After incubation for 1 h, the absorbance was measured at 450 nm with a microplate reader. The control group (A_control_) was set with only cells, and the blank group (A_blank_) was set without cells and protein solution. While the absorbance in the experimental group was set as A_test._ The cytotoxicity was calculated according to the following formula (2).

(2) $$\text{Cell}\;\text{cytotoxicity}\left(\%\;\text{of}\;\text{control}\right)=\frac{A_{\text{test}}-A_{\text{blank}}}{A_{\text{control}}-A_{\text{blank}}}\times 100\%$$

### 2.7 Preparation of photopolymerized K31-PGLa hydrogels

The mixture of K31-PGLa fusion protein and Polyethylene Diacrylate (PEGDA) with a total concentration of 500 μg/mL (Protein concentration determined by BCA reagent kit) were prepared with different mass ratios of 100/0, 94/6, 90/10, 86/14 and 0/100. Then 1% of blue light initiator lithium phenyl-2, 4, 6-trimethylbenzoylphosphinate (LAP), was filtered by 0.22 μm filter membrane and added into the mixture. The solution was mixed in different proportions and polymerized under 405 nm blue light for 0.5–5 min until hydrogels were formed.

### 2.8 Characterizations of photopolymerized K31-PGLa hydrogels

The morphology of freeze-drying photopolymer hydrogels with different mass ratios of K31-PGLa fusion protein to PEGDA was inspected using a Polaron Scanning electron microscopy (SEM) coating system (681HLIPC-691PIPs, USA) at an accelerating voltage of 5.0 kV. For the rheological test, the temperature of the rheometer was controlled at 25 °C. The oscillation frequency was 0.1–100 Hz, and the fixed strain was 5%. *In vitro* degradation of photopolymer hydrogels was investigated within 7 days [45]. Photopolymer hydrogels were weighted and further immersed in PBS at 37 °C. At regular intervals, excess PBS was gently removed, and the samples were weighed. The *in vitro* degradation rate of photopolymer hydrogels was determined as the ratio of a hydrogel mass at a given time point divided by its initial mass. Besides, the swelling behavior of the photopolymer hydrogels with different concentrations was examined at pH 7.4 in PBS buffer at 37 °C. The lyophilized hydrogel was weighed (m_0_) and immersed in PBS, and the soaked hydrogel was weighed (m_t_) every 20 min. The swelling ratio of the hydrogels was calculated as follow formula (3):

(3) Swelling ratio (%) = (m_t_-m_0_)/m_0_ × 100%

### 2.9 In vivo infectious wound healing evaluation

Healthy SD rats (6–8 weeks) were used for infectious wound healing tests and obtained from Laboratory Animal Center of Chongqing Medical University, China). All animal experiments were approved by the Animal Ethics and Laboratory Committee of the Chongqing University. The SD rat was shaved after complete anesthesia, and a square area of 10 mm × 10 mm was marked with a ruler. The labeled epidermis was then cut with sterilized surgical scissors. Inoculate the wound with 200 μL of drug-resistant bacterial solution (OD_600_ = 0.2) 3 times every 1 h. The wounds were then treated with different groups (Ampicillin (AMP, 100 μg/mL), K31-PGLa hydrogel, and AMP/K31-PGLa combination with AMP) and fixed with the Tegaderm film (3 M, St. Paul, MN). Wounds treated with the Tegaderm film were selected as the control group. The hydrogels were changed once a day, and wound treatments and Tegaderm dressing changes were conducted every 3 days. The wound healing process was recorded at predetermined intervals, and the wound area was measured using ImageJ. The wound closure rate of different groups was determined as the ratio of wound area at a given time point divided by its original wound area.

### 2.10 Histological staining

On days 3, 5, 7, and 14 after modeling, newly regenerated tissues and surrounding tissues were harvested and fixed overnight in a 4 °C refrigerator with 10% formalin. Paraffin tissue sections with a thickness of about 4 μm were prepared. According to the steps of the H&E kit, staining and sealing were carried out, and the film was observed and photographed under an optical microscope.

### 2.11 In vivo degradation evaluation

The prepared photopolymer hydrogels (0.5 mL/100 g) were implanted under the skin of SD rats to assess their *in vivo* degradation. The hydrogel degradation was recorded every 7 days, and the residual substances on the surface of the hydrogel were wiped off and weighed to calculate the hydrogel degradation rate. The *in vivo* degradation rate of photopolymer hydrogel was determined as the ratio of a hydrogel mass at a given time point divided by its initial mass.

### 2.12 In vivo biosafety evaluation of photopolymerized K31-PGLa hydrogel

Healthy SD male (6–8 weeks) rats were subcutaneously implantation with 2 g photopolymerized K31-PGLa hydrogel. Blood was collected on days 7, 14 and 21 for ELISA analysis. According to the steps of IL-1β, IL-6 and TNF-α kits, the expressions of three inflammatory factors in serum of each experimental group were detected. The absorbance was measured at 450 nm, the standard curve was drawn, and the contents of inflammatory factors were calculated.

For acute organotoxicity analysis, healthy BABL/c male (6–8 weeks) mice were intraperitoneal injected with 0.2 g of photopolymerized K31-PGLa hydrogels. After 7 days, the major organs of mice were taken for H&E staining.

### 2.13 Immunofluorescence staining

Stained sections were imaged with Leica SP8 confocal microscope. For immunofluorescence staining on rat wound skin tissues, paraffin sections of 10 μm thick were stained with CD3 (Santa Cruz Biotechnology, sc-20047, 1:100 dilution), CD68 (Proteintech, 28058-1-AP, 1:100 dilution) and counterstained with DAPI (Beyotime).

### 2.14 Statistical analysis

All statistical data were obtained from more than three independent samples or three independent replicates, and data were presented as mean ± standard deviation (SD). One-way ANOVA followed by a post-hoc Tukey's multiple comparison test was used. Data were analyzed using SPSS software. The statistical significance level (*p*) was set at < 0.05.

## 3 Results and discussion

### 3.1 K31-PGLa design and synthesis

**Fig. 2:**
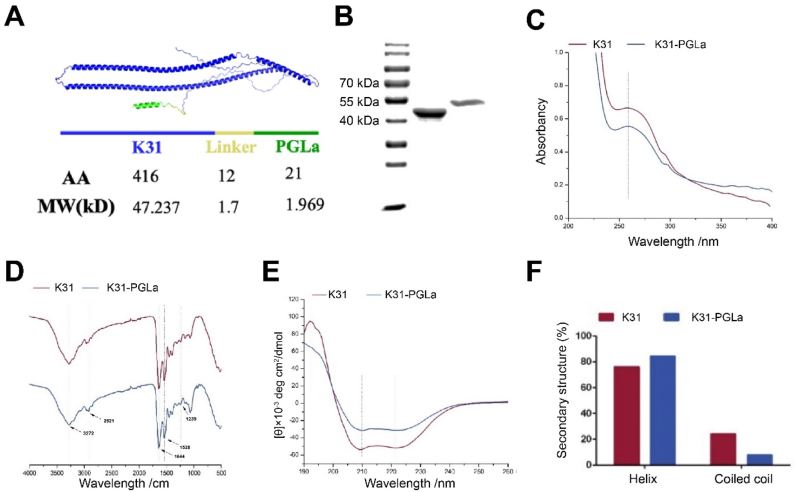
K31-PGLa design and characterizations. (A) Schematic illustration of the K31-PGLa. The gel electrophoresis pattern (B), UV–vis spectra (C), FT-IR spectra (D) and circular dichroism spectra (E) of K31 and K31-PGLa proteins. (F) The secondary structure percent prediction of K31 and K31-PGLa using the BeStSel program.

To obtain K31-PGLa fusion protein material, K31 and K31-PGLa were expressed in an *E. coli* system. K31 is composed of 416 amino acids with a molecular weight of 47.23 kDa. The fusion protein K31-PGLa was composed of keratin K31, a linker peptide and antibacterial peptide PGLa, containing 449 amino acids with a molecular weight of 50.87 kDa (Fig. 2A). According to SDS-polyacrylamide gel electrophoresis (Fig. 2B), the bands were clear and there were no excess stray bands after purification by Ni-beads, indicating high purification efficiency. Molecular weight was between 40 and 55 kDa, which was slightly higher than 47 kDa of K31. The protein molecular weight was close to the predicted size, which was our target protein used for subsequent experiments. The UV–vis absorption spectra of K31 and K31-PGLa were also analyzed as shown in Fig. 2C. The maximum absorption wavelengths of K31 and K31-PGLa were approximately 260 nm, which were similar to the UV–vis spectra of a keratin hydrolysate and recombinant keratins [35]. Keratin solution displayed a wide peak ranging from 250 to 280 nm, and the addition of 8 M urea in the keratin solution to enhance the solubility led to a blue shift in UV–vis spectra. Furthermore, the FT-IR analysis was carried out to investigate the chemical structures of proteins (Fig. 2D). The absorption peaks of the amide I–III band were observed in K31 and K31-PGLa. Typical amide bands correlated in the spectra, several compounds related to the peptide linkage were seen the 3300 cm^−1^ (N–H stretching vibrations), 1700-1600 cm^−1^ (C–O stretching, N–H bending), and 1540-1520 cm^−1^ (C–H stretching vibrations). Vibrations of the C–N stretching and C–O bending at 1220-1300 cm^−1^ were also noted, which represented a typical chemical structure of protein. Moreover, the secondary structures of K31 and K31-PGL were also analyzed by circular dichroism. Two peaks of α helix at 208 nm and 222 nm were detected, indicating that the secondary structures of K31 and K31-PGLa were mainly characterized by α helix structure (Fig. 2E). Next, BeStSel was used to predict the secondary structure composition of recombinant proteins K31 and K31-PGLA. The α helix ratio of K31 and K31-PGLa was 76% and 84.5%, respectively (Fig. 2F). The higher α helix ratio of K31-PGLa could be explained by the fact that PGLa was a α helix-rich protein [46].

### 3.2 K31-PGLa rescues the antibiotic activity of antibiotics against drug-resistant bacteria

**Fig. 3:**
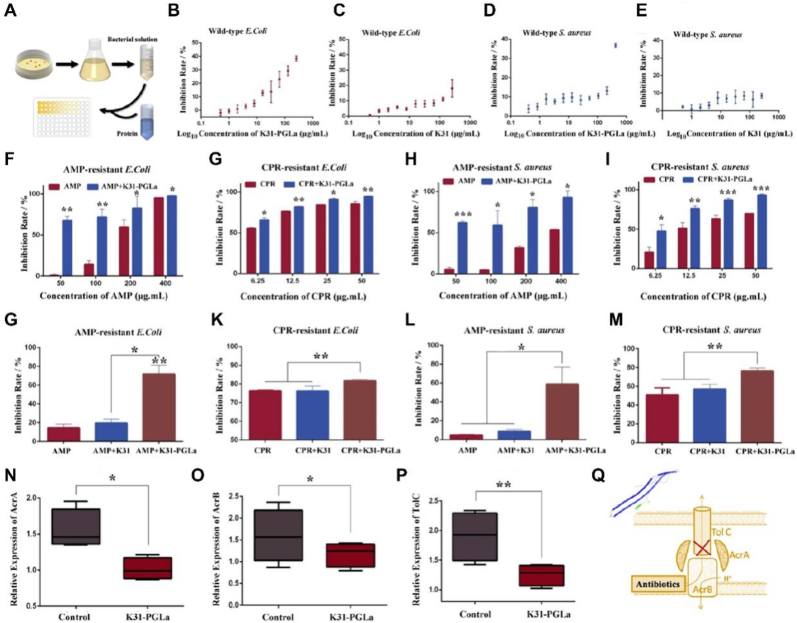
K31-PGLa rescues the antibiotic activity of antibiotics against drug-resistant bacteria. (A) The schematic diagram of minimum inhibitory concentration (MIC) determination of bacteria after being treated with different proteins. (B–E) Antibacterial rate of K31 and K31-PGLa protein against wild type *E. coli* and *S. aureus*, respectively. (F–I) Antibacterial rate of the antibiotic and antibiotic-K31-PGLa combination on drug-resistant strains with various concentrations. (J–M) The effect of antibiotic, antibiotic-K31 and antibiotic-K31-PGLa combinations on drug-resistant strains, and the concentration of AMP and CPR was 100 and 12.5 μg/mL, respectively. (N–P) Relative expression levels of acrA, acrB and tolC in AMP-resistant *E. coli* after treated using K31-PGLa. (Q) The schematic diagram of K31-PGLa rescues the antibiotic activity of antibiotics through the external pump proteins inhabitation. The data are the mean ± SD of each group (n = 3), *p < 0.05, **p < 0.01, ***p < 0.001.

To further investigate the antibiotic resistance reversal efficiency of K31-PGLa, the ampicillin (AMP) or ciprofloxacin (CPR)-resistant *E. coli* and *S. aureus* were obtained by the laboratory evolution method. The antibiotic activities of antibiotics and recombinant proteins against the wild-type and drug-resistant *E. coli* and *S. aureus* were detected by the minimum inhibitory concentrations (MICs) assay (Fig. 3A). Based on the MIC value of the first generation of wild-type strains, we gradually increased drug concentration by 1.5 times, and the AMP or CPR-resistant *E. coli* and *S. aureus* were obtained after 30 days evolution (Fig. S2). Next, we assessed the antibiotic activities of K31 and K31-PGLa against wild-type strains (Fig. 3B–E). The results showed that the inhibition rates of wild-type *E. coli* and *S. aureus* increased with the increase of K31-PGLa concentration. The inhibition rates of *E. coli* and *S. aureus* were less than 40% when K31-PGLa concentration increased to 256 μg/mL. Furthermore, the single K31 showed weak antibiotic activity against wild-type *E. coli* and *S. aureus*, and the inhibition rates of *E. coli* and *S. aureus* were less than 20% and 10%, respectively. Similarly, the K31-PGLa showed unremarkable antibiotic activity, and the antibiotic activity of K31-PGLa was mainly contributed by PGLa. Previous studies verified the antimicrobial activity of PGLa [47,48], but the antimicrobial activity of K31-PGLa was not obvious in our study. The molecular weight of K31 and PLGa was 47.237 kD and 1.969 kD, respectively, so the content of PLGa was relatively low in K31-PLGa protein, although the maximum concentration of K31-PLGa was 1 mg/mL in the antimicrobial test.

We next evaluated whether the K31-PGLa rescued the antibiotic activity of antibiotics against drug-resistant bacteria. The antibacterial effect of antibiotics was significantly reduced against the drug-resistant bacteria after the successful evolution in the laboratory. The inhibition rate of single AMP at 50 μg/mL was approximately 0.25% against AMP-resistant *E. coli*, which was increased to 95.52% at a high concentration of 400 μg/mL (Fig. 3F). Interestingly, the inhibition rate of 50 μg/mL of AMP with 100 μg/mL of K31-PGLa was approximately 63.26% against AMP-resistant *E. coli*, which was 63.01% higher than that treated using single AMP. The same results were observed with the combinations between antibiotics and antibiotic-K31-PGLa against the CPR-resistant *E. coli,* AMP-resistant *S. aureus* and CPR-resistant *S. aureus*. Especially, the combinations of antibiotic and K31-PGLa displayed higher antibiotic activity against drug-resistant bacteria compared to single antibiotic. Additionally, a greater difference in the inhibition rate of antibiotic/K31-PGLa combinations against AMP-resistant bacteria than that treated the CPR-resistant bacteria. The inhibition rate of 50 μg/mL of CPR was 84.70% against CPR-resistant *E. coli*, which was only 9.87% lower compared to 50 μg/mL of CPR combinated with 100 μg/mL of K31-PGLa (Fig. 3G). Moreover, the inhibition rate of 50 μg/mL of AMP was approximately 5.84% against AMP-resistant *S. aureus*, while the inhibition rate increased to 64.46% after treating with 50 μg/mL of AMP and 100 μg/mL of K31-PGLa (Fig. 3H). For the CPR-resistant *S. aureus* inhibition assay, the inhibition rate of 50 μg/mL of CPR was 69.82% against CPR-resistant *S. aureus*, which was 24.11% lower than that treated by 50 μg/mL of CPR and 100 μg/mL of K31-PGLa combination (Fig. 3I). The difference in inhibition rate against AMP-resistant bacteria and CPR-resistant bacteria could be explained by the fact that *E. coli* was more sensitive to CPR compare to AMP. To further confirm that the effect of K31-PGLa on antibiotic resistance reversal would be mainly contributed by PGLa, the inhibition rates of antibiotic, antibiotic/K31 and antibiotic/K31-PGLa against the drug-resistant bacteria were investigated (Fig. 3J-M). The concentrations of AMP and CPR were selected as 100 and 12.5 μg/mL, respectively. No significant difference in inhibition rate was observed between single antibiotic and K31-PGLa against the drug-resistant bacteria, suggesting that the antimicrobial peptide PGLa still maintained good antibacterial activity after fusion with K31, which reversed antibiotic resistance making resistance bacteria drug-sensitive once again. Compared with the materials previously studied, such as ‘8-epidiosbulbin E acetate’ [49] and ‘*Piper longum* fruit extract’ [50], K31-PGLa exhibited a higher reversal efficiency.

One of the reasons for bacterial resistance is the increased synthesis of cell wall surface efflux pump, which excretes intracellular antibiotics out of the cell, and then reduces the intracellular antibiotic concentration [51]. The efflux pump, which is composed of AcrA, AcrB and TolC (encoded by genes acrA, acrB and tolC, respectively), is a trimer protein, in which TolC protein connects to the bacterial outer membrane and AcrB protein connects to bacterial inner membrane [52]. To clarify the effects of K31-PGLa on the expression of acrA, acrB and tolC, fluorescence quantitative PCR was used for verification. The results showed that the expression levels of all three genes were down-regulated by K31-PGLa induction. The genes of acrA, acrB and tolC were reduced by approximately 1.53 times, 1.35 times and 1.52 times, respectively (Fig. 3N-P). We speculated that the increase in the bacteriostatic rate of antibiotics combined with K31-PGLa was attributed to the down-regulation of efflux pump related gene expression and the decrease of efflux pump synthesis, thereby increasing intracellular antibiotic accumulation (Fig. 3Q).

### 3.3 Photopolymerized K31-PGLa hydrogel synthesis and characterizations

**Fig. 4:**
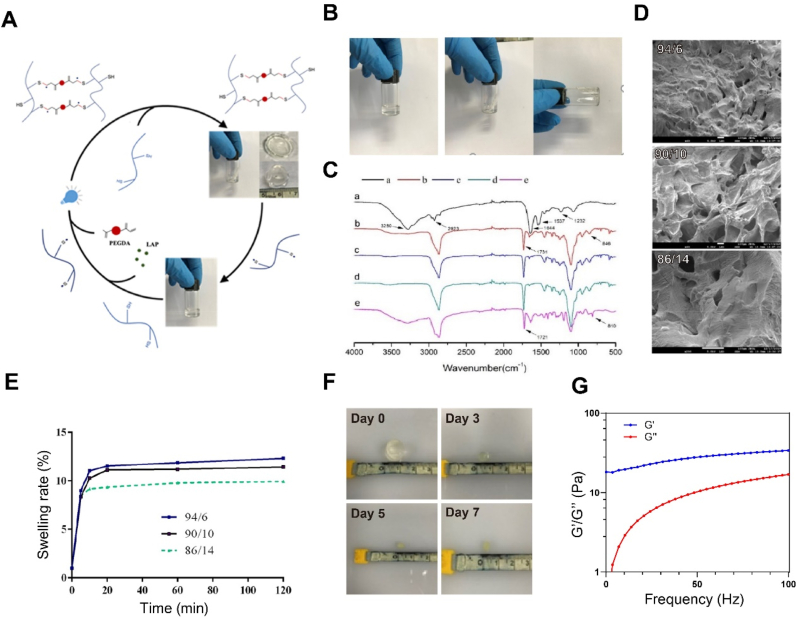
Synthesis and characterizations of photopolymerized K31-PGLa hydrogels. (A) The schematic diagram of photopolymerized K31-PGLa synthesis. (B) Images of hydrogel before and after photopolymerization. (C–F) FT-IR spectra (C), SEM images (D), swelling rate (E) of photopolymerized K31-PGLa hydrogels with different ratios of K31-PGLa to PEGDA. FT-IR spectra of K31-PGLa/PEGDA hydrogel with different ratios: (a) 100/0; (b) 94/6; (c) 90/10; (d) 86/14; (e) 0/100; (F) Photos of *in vitro* degradation of photopolymerized K31-PGLa hydrogel (K31-PGLa/PEGDA = 90/10). (G) Elastic (G′) and Viscous (G″) properties of photopolymerized K31-PGLa hydrogel (K31-PGLa/PEGDA = 90/10). The data are the mean ± SD of each group (n = 3), *p < 0.05, **p < 0.01.

To fabricate K31-PGLa into hydrogels that can fast form gels in situ, we blended fusion protein, crosslinking agent PEGDA and photoinitiator LAP, and irradiated 405 nm blue light to obtain photopolymerized K31-PGLa hydrogels (Fig. 4A). As shown in Fig. 4B, the solution before polymerization was colorless and transparent, and it became a solid hydrogel with fixed shape after 405 nm blue light polymerization. To determine the chemical structures of composited hydrogels, we performed the FT-IR analysis for K31-PGLa hydrogels with different mass ratios of K31-PGLa to PEGDA (100/0, 94/6, 90/10, 84/6, and 0/100) (Fig. 4C). Results showed that new absorption peaks at 1731 cm^−1^ and 846 cm^−1^ belonging to the C]C and –COO– vibrations in the spectra of photopolymerized K31-PGLa hydrogels was noted compared to the spectra of K31-PGLa, indicating that PEGDA was successfully grafted to K31-PGLa protein [[53], [54], [55]]. The surface morphology of photopolymerization hydrogels with different mass ratios of K31-PGLa to PEGDA showed porous structure, and the pore density was decreased with the increase of PEGDA content in SEM (Fig. 4D). Moreover, we also investigated the effect of mass ratios of K31-PGLa to PEGDA on the swelling behavior of photopolymerized K31-PGLa hydrogels (Fig. 4E). The photopolymerized K31-PGLa hydrogels swelled rapidly within 1 h, and the swelling rate of hydrogels was increased with decreasing PEGDA content. The enhancement of crosslinking of K31-PGLa and PEGDA leaded to a decrease in the swelling ratio of hydrogels. The *in vitro* degradation of photopolymerized K31-PGLa hydrogels was assessed (Fig. 4F). The K31-PGLa hydrogels degraded gradually in PBS buffer (pH 7.4), and almost 90% of K31-PGLa hydrogels degraded within 7 days. Besides, rheological analysis results showed that the elastic modulus of photopolymerized K31-PGLa hydrogels was greater than the viscous modulus (Fig. 4G), which was a typical gel property.

### 3.4 Biocompatibility behaviors of photopolymerized K31-PGLa hydrogel

**Fig. 5:**
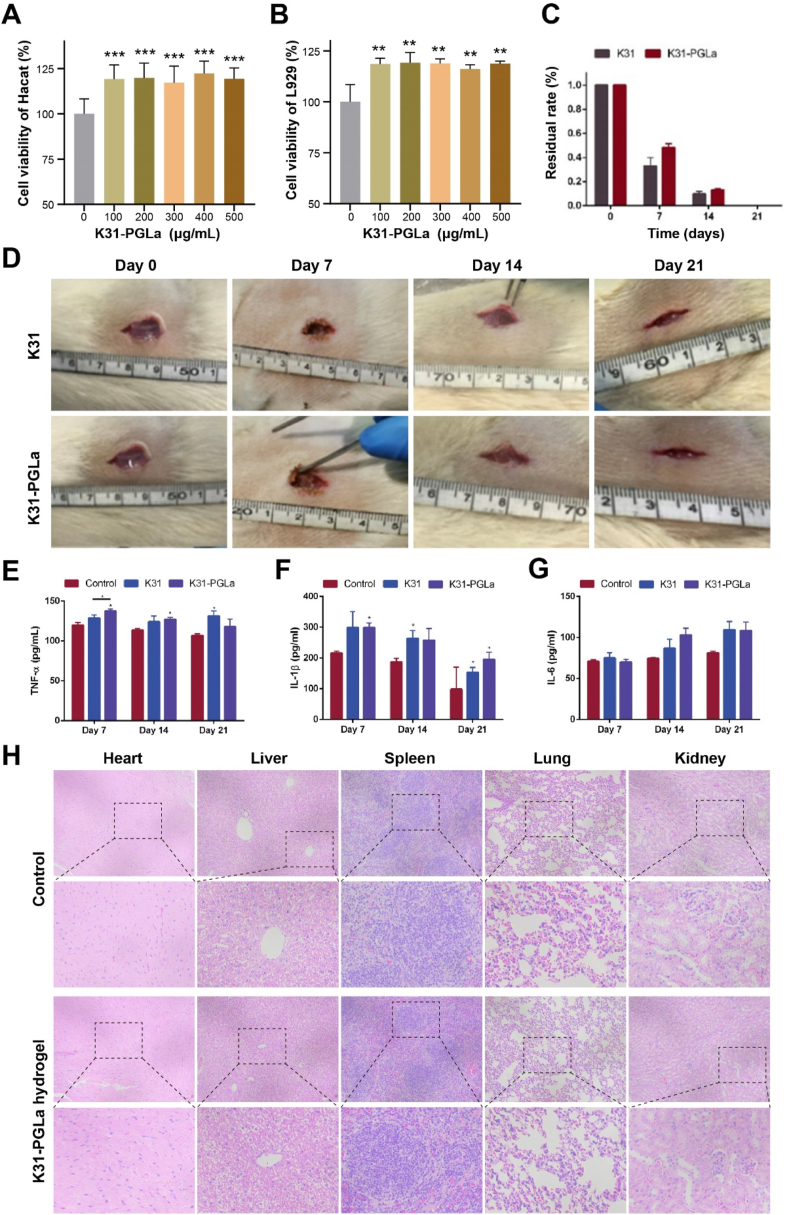
Biocompatibility characterizations of K31-PGLa hydrogel. The effect of different concentrations of K31-PGLa on the proliferation of Hacat cells (A) and L929 cells (B). (C, D) Residual rate (C) and degradation (D) of K31 and K31-PGLa hydrogels within 21 days after implantation under the rat skin. (E–G) Inflammatory factors of TNF-α (E), IL-1β (F) and IL-6 (G) levels in rat serum after implantation of K31 and K31-PGLa hydrogels. (H) H&E staining of major organs of healthy mice after 7 days intraperitoneal injection of K31-PGLa hydrogel. The data are the mean ± SD of each group (n = 3), *p < 0.05, **p < 0.01.

To determine the promotion of cell proliferation and tissue repair for photopolymerized K31-PGLa, Hacat cells and L929 cell lines were selected and CCK8 kit was used to verify the effect of K31-PGLa on cell proliferation (Fig. 5A and B). Compared with the control group, the K31-PGLa group could effectively promote cell proliferation. To obtain the hydrogels with the feature of enough strength and enough space to hold more drugs, we chose the ratio of K31-PGLa/PEGDA = 90/10 for further study. Next, to verify the *in vivo* biodegradability and biocompatibility of the photopolymerized K31-PGLa hydrogels (K31-PGLa/PEGDA = 90/10), we implanted the hydrogels into the subcutaneous tissue of SD rats. The suture line was disconnected every 7 days to observe the degradation and to weight the residual hydrogels. After the implantation of K31 hydrogels and K31-PGLa hydrogels, there was no obvious infection or edema in the rats, indicating that the biocompatibility of the hydrogels was good. Additionally, the degradation rate was calculated after removing the implanted hydrogels and erasing the remaining substances (Fig. 5C). On the first 7 days, the degradation rate of the K31-PGLa hydrogels was faster compared to that of K31 hydrogels (Fig. 5D), and approximately 33% of K31 hydrogels and 48.37% of K31-PGLa hydrogels remained. Moreover, the K31 and K31-PGLa hydrogels were completely degraded at 21 days. Meanwhile, IL-1β, IL-6, and TNF-α are important indicators of inflammatory response [56]. The inflammatory signaling pathway would be activated when an inflammatory response occurs, and the levels of these inflammatory factors would increase. To test whether subcutaneous hydrogel implantation can trigger an inflammatory response in host organisms, the levels of IL-1β, IL-6 and TNF-α in serum of rats were quantitatively determined by ELISA kit (Fig. 5E–G). Compared with the blank group, both the K31 group and the K31-PGLa group caused a slight increase in inflammatory factors after hydrogel implantation. These results suggested that the synthesized K31-PGLa hydrogels did not cause severe inflammatory reactions. Moreover, We also evaluated the acute organotoxicity of the K31-PGLa hydrogels by intraperitoneal injection in mice. H&E staining showed that no significant damage was found in the heart, liver, spleen, lung, kidney and other major organs in the group with hydrogel injection (Fig. 5H). The aforementioned results proved that the photopolymerized K31-PGLa hydrogels had good biocompatibility.

### 3.5 Photopolymerized K31-PGLa hydrogel promotes drug-resistant bacteria-infected wound healing

**Fig. 6:**
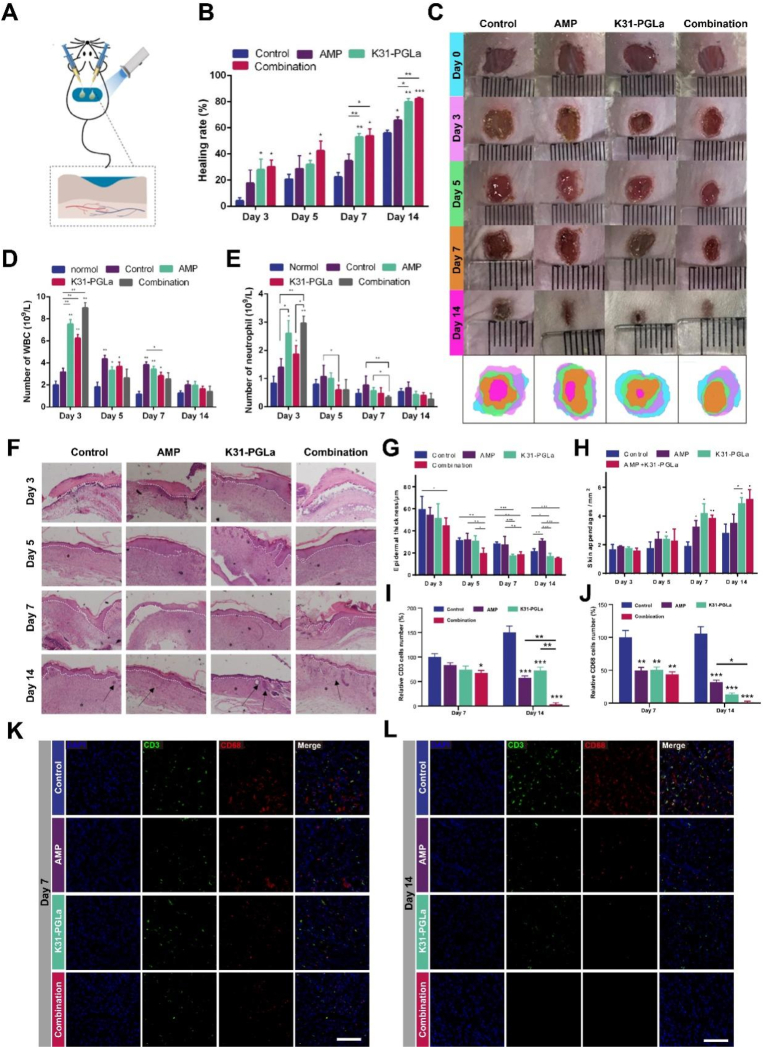
Tissue repair effect of photopolymerized K31-PGLa hydrogel on wounds infected with drug-resistant bacteria in mice. (A) Schematic of AMP/K31-PGLa hydrogels combination treated wound in mice infected with drug-resistant bacteria. (B, C) Quantitative of healing ratio (B) and optical images (C) of wound treated with different groups. (D, E) Count of white blood cells (D) and neutrophils (E) in whole blood of mice infected with AMP-resistant *E. coli* after different treatments. (F–H) H&E staining (F), quantitative statistics of epidermal layer thickness (G) and new skin appendages (H) of corresponding wounds after different treatments. H&E staining shows the boundaries of the epidermal layer (white dashed outline), the formation of blood vessels (black asterisks), and the presence of skin appendages (black arrows). (I–L) Immunofluorescence staining (K,L), quantitative statistics of CD3 + cells (I) and CD68 + cells (J) of corresponding wounds after different treatments. The data are the mean ± SD of each group (n = 3), *p < 0.05, **p < 0.01. Compared with the control group.

The process of wound healing is a series of biological activities of inflammatory cells and tissue repair cells. To investigate the effect of photopolymerized K31-PGLa hydrogels (K31-PGLa/PEGDA = 90/10) on the repair of wounds infected with drug -resistant bacteria, we constructed a full-layer resection wound model in mice with AMP-resistant *E. coli* infection The mice were treated with AMP, K31-PGLa, and combination of AMP and K31-PGLa (Fig. 6A). The results showed that the wound area of each group was gradually reduced with increasing time, and the skin tissue repair speed of the experimental group was faster than that of the control group (Fig. 6B and C). Especially, AMP/K31-PGLa group displayed a significant promotion in wound healing compared to AMP and K31-PGLa groups. On day 5, the wound healing rates of the control group, AMP, K31-PGLa and AMP/K31-PGLa groups were approximately 20.69%, 28.71%, 31.99% and 42.47% respectively on day 5, whereas they increased to 55.98%, 65.78%, 79.86%, and 82.29% respectively on day 14 (Fig. 6B).

White blood cells and neutrophils are responsible for immune function. White blood cells play the role of swallowing foreign bodies and producing antibodies, while neutrophils play the role of sterilization and chemotaxis. When the body encounters bacterial inflammatory infection, tissue injury and surgical trauma, both white blood cells and neutrophils increase. Therefore, to compare the therapeutic effects between the control group and the experimental group, the number of cells in the whole blood of mice was detected (Fig. 6D and E). The results showed that both white blood cells and neutrophils significantly increased after being treated with AMP/K31-PGLa combination compared to other groups, indicating that the bacterial infection model was established successfully. Furthermore, white blood cells and neutrophils in each treatment group decreased gradually with time. The total number of white blood cells returned to normal levels on day 14, while the level of neutrophils returned to normal levels on day 5.

To further determine the repair status of each group, we sectioned the wound skin for H&E staining. The epidermal thickness (Fig. 6F) and density of skin appendages (Fig. 6G) were also quantified. The epidermis (white dotted line outline) became thicker due to hyperplasia and scab, and the AMP/K31-PGLa hydrogels combination led to a significant decrease in the epidermal thickness compared to the AMP group and K31-PGLa hydrogels group. Besides, a significant increase in skin appendages (black arrows) was observed in the AMP/K31-PGLa hydrogels combination group compared to the AMP group and control group. Taken together, K31-PGLa could rescue the antibiotic activity of AMP against AMP-resistant *E. coli*, and promote wound healing infected with drug-resistant bacteria in mice.

To evaluate the infection status of the wound, we performed immune cell staining by immunofluorescence staining on the wound skin tissue (Fig. 6K and L). On the seventh day, the AMP/K31-PGLa hydrogels combination led to a significant decrease in T cells (CD3^+^ cell) number compared to the control group. On the 14th day, the number of T cells in the other three groups all showed a significant decrease, while the AMP/K31-PGLa hydrogels combination group showed the highest degree of decrease (Fig. 6I). For macrophages (CD68^+^ cells), the number of macrophages in AMP group, K31-PGLa hydrogel group and AMP/K31-PGLa hydrogels combination group all decreased significantly on the 7th day compared to the control group. On the 14th day, the number of macrophages in the AMP group and K31-PGLa hydrogels group continued to decrease compared to the control group, whereas almost no macrophage was observed in the combined treatment group (Fig. 6J). Summarily, K31-PGLa could rescue the infection of drug-resistant wounds, especially when combined with AMP, the healing process was significantly sped up.

## 4 Conclusion

Overall, we reported the photopolymerized keratin-PGLa hydrogels for antibiotic resistance reversal and enhancement of infectious wound healing. The K31- PGLa displayed an unremarkable antibiotic activity against wild-type *E. coli* and *S. aureus*, but the outstanding synergistic effect with commercial antibiotics against drug-resistant bacteria by down-regulating the synthesis genes of efflux pump. Moreover, the photopolymerized K31-PGLa hydrogels were developed as a suitable means of wound healing therapy, which exhibited good biocompatibility and wound healing properties infected with drug-resistant bacteria. However, further research will be needed to deal with more complex wound infections, such as other types of bacterial or fungal infections, and wound infections in patients with diabetes. Given the serious challenges in infectious wound management and the embarrassment of new antibiotics development, our work provided a promising alternative strategy for the treatment of drug-resistant bacteria infection wounds.

## Credit author statement

**Changfa Sun:** Conceptualization Methodology, Formal analysis, Writing – original draft, Visualization. **Wenjie Liu:** Conceptualization, Methodology, Formal analysis. **Lili Wang, Run Meng:** Investigation, Writing. **Jia Deng:** Conceptualization, Methodology, Formal analysis. **Rui Qing:** Conceptualization, Writing – review & editing, Supervision. **Bochu Wang, Shilei Hao:** Conceptualization, Writing – review & editing, Supervision.

## Declaration of competing interest

The authors declare that they have no known competing financial interests or personal relationships that could have appeared to influence the work reported in this paper.

## Data Availability

Data will be made available on request.
