# Supplementary material for: Photopolymerized keratin-PGLa hydrogels for antibiotic resistance reversal and enhancement of infectious wound healing
Source: Mater Today Bio. 2023 Sep 23;23:100807. doi: 10.1016/j.mtbio.2023.100807 (PMC10558788; doi:10.1016/j.mtbio.2023.100807)
Supplement: Multimedia component 1 [file mmc1.docx]

**Photopolymerized keratin-PGLa hydrogels for antibiotic resistance reversal and enhancement of infectious wound healing**

Changfa Sun ^a, 1^, Wenjie Liu ^a, 1^, Lili Wang ^a^, Run Meng ^a^, Jia Deng ^b,*^, Rui Qing ^c^, Bochu Wang ^a, *^, Shilei Hao ^a,^ *

^a^ Key Laboratory of Biorheological Science and Technology, Ministry of Education, College of Bioengineering, Chongqing University, Chongqing, 400030, China.

^b^ College of Environment and Resources, Chongqing Technology and Business University, Chongqing, 400067, China.

^c^ State Key Laboratory of Microbial Metabolism, School of Life Sciences and Biotechnology, Shanghai Jiao Tong University, Shanghai 200240, China

*Corresponding author Email：

[jiadeng2011@hotmail.com](mailto:jiadeng2011@hotmail.com) (J. Deng)

[wangbc2000@126.com](mailto:wangbc2000@126.com) (B. Wang)

shilei_hao@cqu.edu.cn (S. Hao)

^1^ Changfa Sun and Wenjie Liu contributed equally to this work.

**Appendix A. Supplementary data**


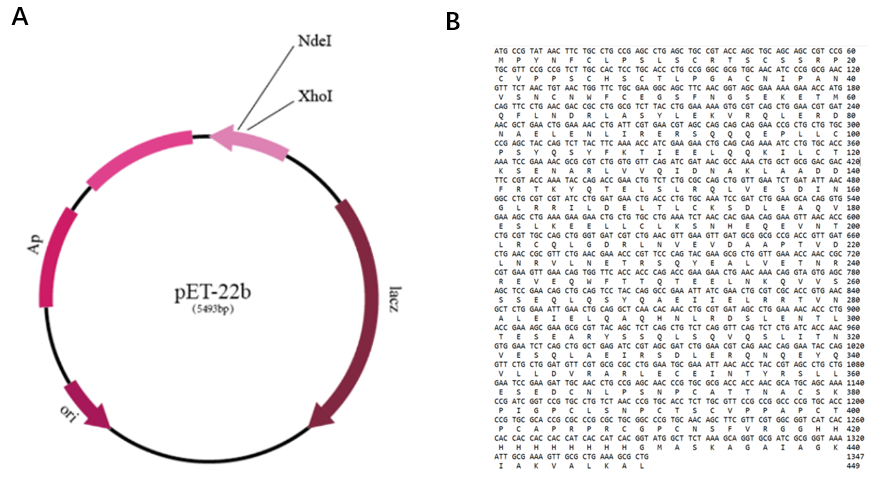


Fig. S1. (A) Construction of plasmid vector. (B) Amino acid and gene sequence of fusion protein K31-PGLa

**
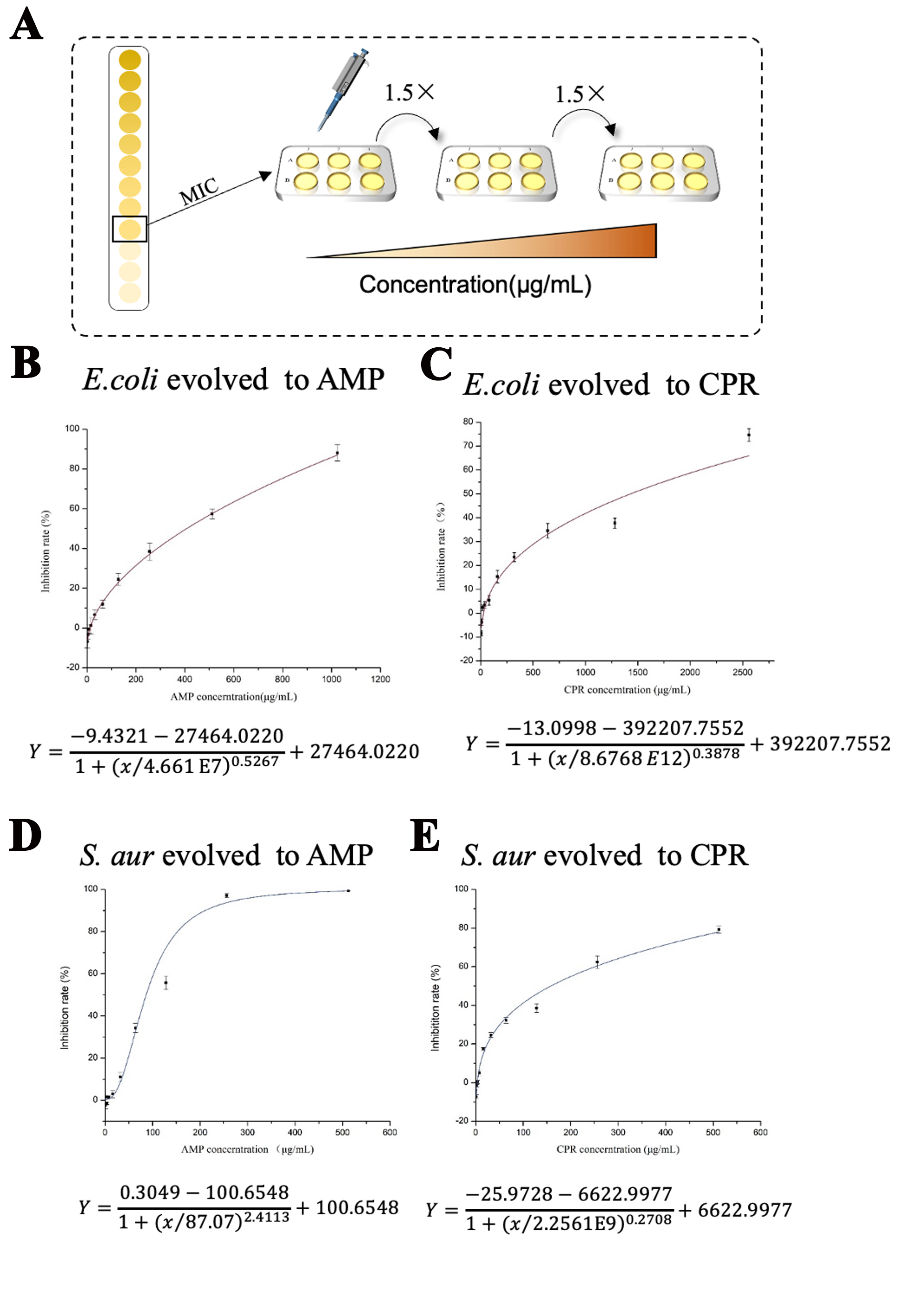
**

Fig. S2. (A) The evolution flow chart of laboratory resistant bacteria. (B, C) *E. coli* tolerance after 30 days of laboratory evolution. Dose-response curve for (B) AMP and (C) CPR. (D, E) *S. aur* tolerance after 30 days of laboratory evolution. Dose-response curve for (D)AMP and (E) CPR.

**Table S1. Primer sequences used for quantitative real-time PCR analysis.**

| Gene name | Primer sequence |
| --- | --- |
| marR | F:TGGATATTACCGCGGCACAG |
|  | R:GCCTTTACAGACCAGGCGAT |
| acrA | F:CGACAAACAGGCCCAACAAG |
|  | R:CGCTTCAGGATAATCCCGCT |
| acrB | F:CGAAGATTGAGCTGGGTGGT |
|  | R:TTTTCAGACCCGACGGGAAG |
| TolC | F:GAAAAACGCAACCTGTCGCT |
|  | R:ACGGGTTTTCGAACCGCTAT |
| 16S | F:AGGCCCGAAACTGACGATTT |
|  | R:CATGTCGGCAATGGCATCAG |
